# A novel integration of Hodrick–Prescott filter (Hp-filter) and wavelet transform (WT) with optimize support vector machine (PSO-SVM) in predicting solar radiation

**DOI:** 10.1038/s41598-024-82950-2

**Published:** 2025-04-25

**Authors:** Shuvendu Pal Shuvo, Shirshendu Pal Shibazee, Goutam Paul, Mitaly Paul Mita, Chaitee Das, Konika Malakar

**Affiliations:** 1 Department of Civil Engineering, Khulna University of Engineering and Technology, Khulna, Bangladesh; 2 Department of Electrical and Electronic Engineering, Bangladesh University of Engineering and Technology, Dhaka, Bangladesh; 3 Department of Pharmacy, Bangabandhu Sheikh Mujibur Rahman Science and Technology University, Gopalganj, Bangladesh; 4 Department of Economics, Khulna University, Khulna, Bangladesh; 5 Department of Materials Science and Engineering, Khulna University of Engineering and Technology, Khulna, Bangladesh; 6 Department of Environmental Science and Resource Management, Mawlana Bhashani Science and Technology University, Dhaka, Bangladesh

**Keywords:** Solar radiation, Support vector machine (SVM), Hodrick Prescott Filter (HP-Filter), Discrete wavelet transform (DWT), Climate sciences, Environmental sciences

## Abstract

Previous research has shown that predicting solar radiation is a challenging issue due to highly nonlinear and noisy climate data. Various hybrid approaches have been applied earlier for solar radiation prediction, which integrates the Wavelet Transform with various Machine Learning models. This research, therefore, intends to further improve the performance of these existing hybrid models. To address the limitations in handling nonlinear and noisy climate patterns, this study proposes a multi-hybrid model for accurately predicting solar radiation that incorporates the Hodrick–Prescott Filter (HP-Filter), Discrete Wavelet Transform (DWT), and Support Vector Machine (SVM). The collected data from the Bangladesh Meteorological Department for two different geological locations in Bangladesh, namely Dhaka and Chittagong, is divided into three categories for modeling: 70% for training, 15% for validation, and 15% for testing, whereas the model hyper-parameters of the SVM were optimized using the Particle Swarm Optimization algorithm. The proposed approach applies the Hodrick–Prescott Filter before analyzing DWT to strengthen the SVM model’s ability to capture complicated climate patterns in great detail and also make the model more precise and reliable. Several performance metrics, such as Mean Squared Error (MSE), Root Mean Squared Error, Mean Absolute Error, Mean Absolute Percentage Error, and Coefficient of Determination (R^2^), were considered for model evaluation. The results showed that it improves upon traditional SVM by 99.76% and 99.77% and hybrid DWT-SVM by 39% and 57% in terms of MSE reduction at Dhaka and Chittagong, respectively. R^2^ also improved by 49% and 54% over traditional SVM and by 4.40% and 3.16% over hybrid DWT-SVM model. The model well captures the complex nonlinear trend existing in solar radiation; thus, it shows its potential to be applied to other regions for efficient prediction of solar radiation.

## Introduction

Bangladesh is an overpopulated country with 1% yearly population growth. This, along with the country’s growing economy, results in an average annual rise in energy demand of 4.68%. Bangladesh currently gets 99% of its energy from fossil fuels (Report 2021), but by 2030 and 2040, the balance could change in preference to sources of renewable energy. Solar electricity will play an important role in achieving these goals^1^. The total power generation capacity as of date is about 31,452 MW, and the maximum generation in 2024 stood at 16,233 MW. The contribution of renewable energy sources among these is a meagre 1373.81 MW. Solar energy contributes 1079.82 MW, which forms only 6.25% of the net power generation^2,3^. Thus, it is very important to expand the use of solar power compared to fossil fuels like coal, which are non-renewable and environmental pollutants. In that direction, a solar radiation prediction model with high accuracy for Bangladesh is of prime importance. Accurate prediction of solar radiation is hence essential for scheduling and preparing solar energy system optimization in order to ensure the most effective energy production and grid integration^4^. The predictions will thus allow better management of solar power variability, leading to a stabilized and reliable energy supply^5^. This is therefore very important in reducing dependency on fossil fuels and increasingly embracing renewable sources of energy in the move towards environmental sustainability and climate change mitigation^6^. Furthermore, accurate solar radiation forecasts support agricultural planning and enhance crop management and irrigation practices by providing insights into potential sunlight exposure. They are also important for weather forecasting, climate research, and various industrial applications that require working efficiently, as solar exposure affects operations^7^.

Multiple methodologies are used to predict global solar radiation across various time horizons. Overall, the approaches can be divided into physical, process-based, and data-driven approaches^8–11^. Previous research has focused on time series models for solar radiation forecast such as Seasonal Autoregressive Integrated Moving Average (SARIMA)^12^, Autoregressive Integrated Moving Average (ARIMA)^13,14^, and Autoregressive Moving Average (ARMA)^14^. Handling such complex and noisy data using these time series models becomes difficult. Recent Machine Learning (ML) advances have overcome these challenges, allowing the identification of complex patterns that exist in time series data and enhancing prediction accuracy^14^. Several ML algorithms have been utilized earlier for solar radiation prediction, most of which have gained considerable success: Artificial Neural Networks^15^, Support Vector Machines^16^, Long Short-Term Memory Networks^17^, Adaptive Neuro-Fuzzy Inference Systems^18^, and Decision Trees^19^. These methods have been used to show good accuracy in the solar radiation forecast by capturing complex patterns and relationships within the data. Hybrid models that combine traditional time series and machine learning methods, as well as ML ensembles, have also been investigated to try to deal with the nonlinear and noisy characteristics of solar radiation in a way that can improve prediction performance^20–23^. The hybrid techniques find it difficult to minimize the error due to highly nonlinear and noisy data; therefore, signal processing-based denoising approaches have gained momentum in the recent past. For example, advanced methods like Multivariate Empirical Mode Decomposition with Ant Colony Optimization (MEMD-ACO), Ensemble Empirical Mode Decomposition (EEMD), and Variational Mode Decomposition (VMD) have been used with models like Random Forest (RF) and Regression Ensemble (RE) in countries like Australia and China. These studies have shown error metrics, such as Root Mean Square Error (RMSE), as low as 0.41 and Relative Root Mean Square Error (RRMSE) between 4.45 and 9.35%^24–27^. Other combinations, like Principal Component Analysis (PCA) with Artificial Neural Network (ANN) in India, reported an RMSE of 12.06^28^.

The Wavelet Transform (WT), which is used in this study has recently been interpreted in the literature as a very efficient data pre-processing tool that is expected to increase the performance of any ML model to a great extent, irrespective of region. Table 1 illustrates the recent studies based on using the WT during solar radiation prediction. For example, Salisu et al. integrated WT with an Adaptive Neuro-Fuzzy Inference System (ANFIS) and obtained a Mean Absolute Percentage Error (MAPE) of 2% in Nigeria^29^, while Abdullah et al. achieved a MAPE of 1.40% using WT with a Multilayer Perception (MLP) in Malaysia^30^. In the UAE, WT was used with multiple models such as General Regression Neural Network (GRNN), Nonlinear Autoregressive Exogenous Model (NARX), ANFIS, and MLP by Hussain and Al Ali (2017) to obtain RMSE values from 2.78 to 4.10^31^. Deo et al. (2016) achieved a MAPE of 12.66 by combining WT with the Support Vector Machine (SVM) in Brisbane^32^. More recent research conducted by Küçüktopçu et al. (2024) in Turkey used WT in combination with SVR, RF, and K-Nearest Neighbors (KNN), returning an RMSE of 2.174, 2.223, and 2.436, respectively^33^. All of these studies underline the effectiveness of WT in enhancing model accuracy under different scenarios. Wavelet Transform (WT) has also been used with models like MLP, Recurrent Neural Network (RNN), ANN, and Gaussian Process Regression (GPR) in many developing countries, including China, India, Spain, and the UAE, showing the performance metrics of Mean Absolute Error (MAE) values of 0.72 and 9.6, and RMSE values in a range of 17.2 to [38.9–28.7]^34–37^. WT, combined with SVM, had some really promising results in Iran, with a MAPE as low as 6.99% in the year 2014 and an RMSE of 0.69 in the year 2015, so the results were very accurate^38,39^. In Algeria, WT was combined with GPR in the year 2021 and returned a moderate RMSE of 1.81^40^. The combination of WT with ANN in Turkey returned a very high RMSE of 19.23 in 2020^41^.

**Table 1:** Wavelet transform-based existing solar radiation prediction model.

Data pre-processing technique	Method	Location	Model performance matrix	Year	References
Wavelet transform (WT)	ANFIS	Nigeria	MAPE = 2%	2018	^29^
Wavelet transform (WT)	MLP	Malaysia	MAPE = 1.40%	2018	^30^
Wavelet transform (WT)	GRNN	UAE	RMSE = 2.78	2017	^31^
Wavelet transform (WT)	NARX	UAE	RMSE = 3.29	2017	^31^
Wavelet transform (WT)	ANFIS	UAE	RMSE = 4.10	2017	^31^
Wavelet transform (WT)	MLP	UAE	RMSE = 3.89	2017	^31^
Wavelet transform (WT)	SVM	Brisbane	MAPE = 12.66	2016	^32^
Wavelet transform (WT)	SVR	Türkiye	RMSE = 2.174	2024	^33^
Wavelet transform (WT)	RF	Türkiye	RMSE = 2.223	2024	^33^
Wavelet transform (WT)	KNN	Türkiye	RMSE = 2.436	2024	^33^
Wavelet transform (WT)	SVM	Iran	MAPE = 6.99%	2014	^38^
Wavelet transform (WT)	SVM	Iran	RMSE = 0.69	2015	^39^
Wavelet transform (WT)	GPR	Algeria	RMSE = 1.81	2021	^40^
Wavelet transform (WT)	ANN	Turkey	RMSE = 19.23	2020	^41^
Wavelet transform (WT)	ANN	Iraq	RMSE = 2.84 – 3.47	2023	^42^
Wavelet transform (WT)	SVM	Iraq	RMSE = 2.45–3.92	2023	^42^
Wavelet transform (WT)	ANFIS	Iraq	RMSE = 2.64—3.29	2023	^42^
Wavelet transform (WT)	BILSTM	Turkey	RMSE = 53.20	2024	^43^
Wavelet transform (WT)	LSTM	Turkey	RMSE = 67.43	2024	^43^
Wavelet transform (WT)	RNN	Turkey	RMSE = 67.96	2024	^43^
Wavelet transform (WT)	ANN	Turkey	RMSE = 54,38–78.40	2023	^44^
Wavelet transform (WT)	LightGBM (LGB)	Greece	RMSE = 135.68	2024	^45^

Techniques like WT-ANN, WT-SVM, and WT-ANFIS in Iraq have quite low RMSE values ranging from 2.45 to 3.92, indicating high predictive accuracy for different studies by Anupong et al.^42^. In contrast, deep learning architecture models were applied in Turkey with phenomenally higher values of RMSE. WT-BILSTM has reached up to 53.20, and WT-RNN reached 67.96, according to Çevik Bektaş and Altaş^43^. A WT-ANN model for Turkey studied by Kaysal and Hocaoğlu (2023) yielded a broader range of RMSE values from 54.38 to 78.40^44^. Further, the LGB implemented by Blazakis et al. (2024) in Greece returns a very high RMSE of 135.68^45^. In such hybrid approaches, optimization algorithms make a major contribution to the results of the model. Reviewing the work by Álvarez-Alvarado, it can be seen that various optimization techniques, such as Particle Swarm Optimization (PSO) and Genetic Algorithm (GA), have shown promising results when coupled with the SVM model^16^. Proper selection of an optimization technique is very important and enhances the accuracy of the model by fine-tuning the parameters for best performance. Harris Hawks Optimization (HHO) is one of the meta-heuristics algorithms developed recently. Although these have shown promise in many different arenas, applications for the estimation of solar radiation are developing. Ramalingam created a model of prediction of solar radiation using Harris Hawks Optimization that achieved high accuracy with an RMSE value of 0.0801–0.23^46^.

While hybrid techniques generally improve the accuracy of solar radiation prediction models, different investigations have found that they may occasionally generate higher errors. This is a call to action for more accurate models that can effectively minimize errors and noise while also allowing for more dependable forecasts. There is a need for recent advances that address current limitations to increase forecast accuracy. This research has integrated a double-step noise and feature reduction method for enhancing the accuracy of solar radiation prediction. This improved methodology aims to provide a way to cope with the problems brought by geological features and other variables that impact this phenomenon, and it will eventually result in a more robust and precise model compared to existing techniques.

The novelty of the research consists in developing a model that incorporates the Hodrick–Prescott Filter (HP-Filter) that ensures accurate smoothing of the data, a Discrete Wavelet Transform (DWT) for noise reduction in noisy data, and a Particle Swarm Optimization (PSO)-based Support Vector Machine (SVM) for better prediction accuracy. All these filters together put more force into the performance of the entire model by effectively removing noise and capturing the fundamental trends, which culminates in more reliable solar radiation forecasts. Table 1 summarizes some contributions that used only one Wavelet Transform combined with many machine learning and deep learning models, resulting in larger errors. However, there is a research gap in investigating the effect of using a filtering approach before applying the Wavelet Transform. Furthermore, there has been a lack of studies that use the DWT, HP-Filter and SVM to predict solar radiation in Bangladesh. In this study, the Hodrick-Prescott Filter (HP-Filter) is combined with the Wavelet Transform (Discrete Wavelet Transform) in the Support Vector Machine (SVM) model. It could be a novel approach to integrate these approaches. As a developing country, Bangladesh needs accurate and reliable models for its solar radiation modeling, which is crucial for the exploitation of solar energy resources. In this paper, two major geographical locations in the country are considered: Dhaka and Chittagong. The proposed model would mainly aim at achieving high accuracy compared to the previously existing hybrid and traditional methodologies. Previously, a Wavelet Transform-based model was employed in multiple studies. In this study, the HP-Filter is used before the DWT as a filtering method. This strategy aims to reduce the level of noise and error in the WT-based model. To assess the effectiveness of the proposed novel technique, a hybrid Wavelet Transform-based model and a lag-time-based traditional model have been generated for estimating solar radiation.

## Theoretical overview

The model development process in this research was accomplished by bringing together three distinct but complementary materials: the application of a machine learning algorithm, specifically the Support Vector Machine; the use of Discrete Wavelet Transforms for data preprocessing and feature extraction; and the application of the Hodrick–Prescott Filter in signal filtering and noise reduction. This study investigated the synergies between such advanced methodologies to achieve more precise and dependable results and to push the predictive modeling and signal processing fields forward.

### Support vector machine (SVM)

**Fig. 1:**
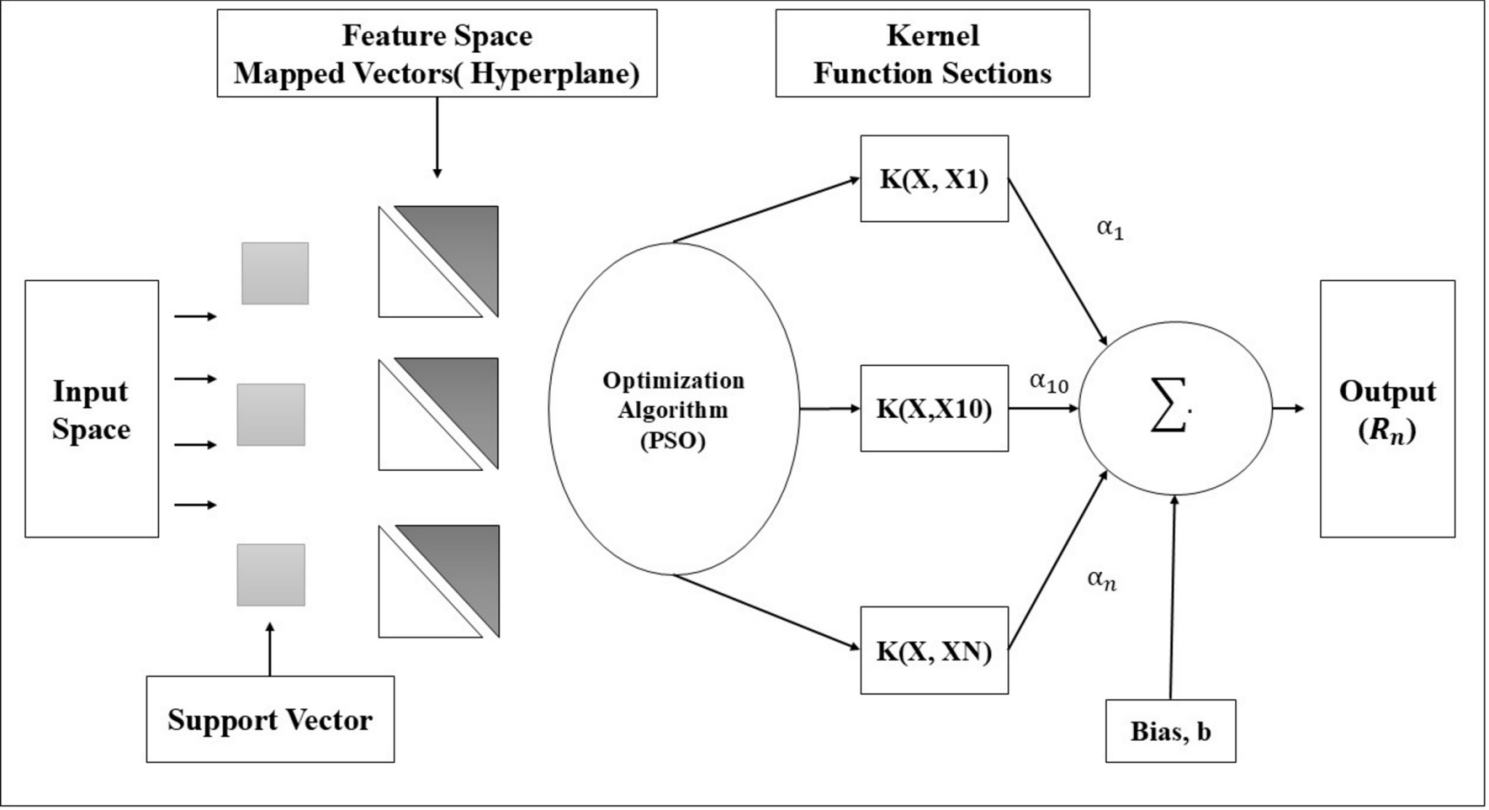
Basic working principle of support vector machine.

Support Vector Machine (SVM) is a very robust and famous model of machine learning, built for classification and regression tasks^47^. The algorithm constructs a hyperplane that maximizes the margin between the closest data points of each class, known as support vectors, in the feature space. SVM works very well in high-dimensional spaces and can treat linear and nonlinear relationships^48^. In regression tasks, SVM is renamed Support Vector Regression. Compared to other regression models, which directly minimize the error, SVR attempts to fit a model that is as far away from the true target values as feasible while maintaining the errors under a certain threshold^49^. This will ensure that the model is not overly sensitive to outliers, resulting in a more reliable prediction framework. The basic SVM structure is illustrated in Fig. 1. The SVM is defined as Eq. (1)^32^

1 $${\text{R}}_{{\text{n}}} \equiv {\text{y }} = {\text{f}}\left( {\text{x}} \right) = {\text{w}} \cdot \left( {\text{x}} \right) + {\text{b}}$$

where w is the weighted vector, b is the constant, and $\varphi$ (x) denotes a mapping function used for mapping in feature space. Coefficients w and b are estimated as follows by minimization Eqs. (2) and (3):

2 $${\text{R}}_{{{\text{reg}}}} \left( {\text{f}} \right) = {\text{C}}\frac{1}{N}\mathop \sum \limits_{{i = 1}}^{N} L_{\varepsilon } ({\text{f }}(X_{i} ),y_{i} ) + ~\frac{1}{2}\left\| {w^{2} } \right\|$$

3 $$L_{\varepsilon } \left( {{\text{f}}\left( {\text{x}} \right) - {\text{y}}} \right){\text{ }} = \left\{ {\begin{array}{*{20}l} {\left| {f\left( x \right) - y} \right| - \varepsilon } \hfill & {for\;~\left| {f\left( x \right)y} \right| \ge \varepsilon } \hfill \\ 0 \hfill & {otherwise} \hfill \\ \end{array} } \right.$$

It has some major components involved in the working of the SVM model. The elements, namely, C and $\varepsilon$, are fed into the model. The first term of the equation, ${L}_{\varepsilon }($(f(x_i_), y_i_)), is referred to as the $\varepsilon -$ insensitive loss function. It provides what might be called a “tube” around the decision boundary within which errors are not penalized. This constitutes a very critical component controlling a tradeoff between model complexity and margin width. Within this context, the target variable y_i_, namely solar radiation in the i-th period. The method uses input features to predict, so the SVM model is an excellent tool for tasks like the forecasting of solar radiation levels over time.

This function indicates errors below $$\varepsilon$$ are not penalized, while the term C $$\frac{1}{N}$$ $$\sum_{i=1}^{N}{L}_{\varepsilon }$$(f ($${X}_{i}$$), $${y}_{i})$$ represents the SVM model’s empirical error. The term $$\frac{1}{2}$$ $${\Vert w\Vert }^{2}$$ measures the function’s smoothness, C evaluates the link between empirical risk and smoothness, and the positive slack variables $$\xi$$ and ξ^*^ represent the distance between actual values and corresponding boundary values in $$\varepsilon -tube$$ Eq. 2 is transformed as follows in Eqs. (4) and (5):

4 $${\text{Minimize}}\;\frac{1}{2}\left\| w \right\|^{2} + {\text{C}}\frac{1}{N}\mathop \sum \limits_{{i = 1}}^{N} \left( {\xi _{i} + \xi _{i}^{*} } \right)$$

5 $${\text{Subjected }}\;{\text{to}}\left\{ {\begin{array}{*{20}l} {\left| {y_{i} - (\left( {w,x_{i} } \right) + b} \right| \ge \varepsilon + \xi_{i} } \hfill \\ {\left( {w,x_{i} + b - y_{i} \le \varepsilon + \xi_{i}^{*} } \right)} \hfill \\ {\xi_{i} ,\xi_{i}^{*} \ge 0 } \hfill \\ \end{array} } \right.$$

After applying the Lagrangian and optimal conditions, a non-linear regression function is obtained (Eq. 6):

6 $${\text{f}}\left( {\text{x}} \right) = \mathop \sum \limits_{i = 1}^{l} (\alpha_{i} - \alpha_{i}^{*} )k\left( {x_{i} ,x} \right) + b$$

where ${\alpha }_{i}$ and ${\alpha }_{i}^{*}$ are the Lagrange multipliers. The $k({x}_{i},x)$ is the kernel function that describes the inner product in D-dimension feature space viz Eq. (7):

7 $${\text{k }}\left( {{\text{x}},{\text{ x}}_{{\text{i}}} } \right) \, = \mathop \sum \limits_{i = 1}^{D} \varphi_{j } \left( x \right) \varphi_{i} \left( y \right)$$

In this study three kernel functions are considered like linear kernel function (Eq. 8), Gaussian RBF kernel (Eq. 9), and Polynomial kernel (Eq. 10).

8 $${\text{Linear}}:{\text{ K}}(\omega , b) = \omega^{T} {\text{x}} + {\text{b}}$$

9 $${\text{Polynomial}}:{\text{ K }}(\omega , x) = \left( {\gamma \omega^{T} {\text{x }} + {\text{ b}}} \right){ }^{N}$$

10 $${\text{Gaussian RBF}}:{\text{ K }}(\omega , x) = {\text{exp }}( - \gamma \left\| {x_{i} - x_{j} } \right\| ^{{\text{n}}} )$$

### Particle swarm optimization (PSO) algorithm

Optimizing the Kernel function and the hyperparameters, such as regularization parameter (C), is very important to the model. This work, therefore, applies Particle Swarm Optimization (PSO) with the view of finding the best-fit parameters for the SVM model efficiently. PSO is preferred for optimizing the parameters of SVMs since it is simple, adaptive, and usually performs very well in complex search spaces^50^. Unlike such exhaustive methods as Grid Search or such algorithms that require such complex operations as Genetic Algorithm (GA)^51^, PSO only updates the position and velocity of particles, making its implementation very easy. PSO balances the exploratory and exploitative stages by guiding particles toward the best-known solutions; hence, fast convergence is possible even for non-convex spaces where other techniques, including Gradient Descent, will often falter^52^. It is inherently parallelizable; hence, efficient evaluation of SVM parameters can be done on big, high-dimensional spaces. This adaptability facilitates PSO in finding the optimal values of important SVM parameters, such as Kernel type and regularization (C), that produce models with lower errors and improved generalization. This is a selection of parameters improved by PSO, which acts like the social behavior of particles, with each particle representing a candidate set of parameters of SVMs. The positions of the particles are iteratively adjusted by the algorithm to balance the individual search represented by the best-known position of each particle with the collective one taken as the best-known position of the swarm to obtain an improved solution. Thus, PSO adapts dynamically to guide the particles toward optimal configurations. In this way, the search space of a Linear, Polynomial, and Gaussian RBF is defined through the range of regularization parameters^18^. The objective function to minimize can be an error rate on a validation set or cross-validation accuracy. That function evaluates how well the SVM with given parameters is performing. Randomly initialize the position of particles within the defined search space. After that, train an SVM using these parameters given by every particle and check the value of the objective function. The updated velocity and position of each particle are given by Eqs. (11) and (12) of PSO:

11 $$v_{i} \left( {t + 1} \right) \, = \, \omega v_{i} \left( t \right) \, + \, c1r1\left( {p_{i} - x_{i} } \right) \, + \, c2r2\left( {g - x_{i} } \right){\text{ v}}_{{\text{i}}} \left( {{\text{t}} + 1} \right) \, = \, \upomega {\text{v}}_{{\text{i}}} \left( {\text{t}} \right) \, + {\text{ c}}1{\text{r}}1\left( {{\text{p}}_{{\text{i}}} - {\text{x}}_{{\text{i}}} } \right) \, + {\text{ c}}2{\text{r}}2\left( {{\text{g}} - {\text{x}}_{{\text{i}}} } \right)$$

12 $$x_{i} \left( {t + 1} \right) \, = x_{i} \left( t \right) + v_{i} \left( {t + 1} \right){\text{ x}}_{{\text{i}}} \left( {{\text{t}} + 1} \right) \, = {\text{x}}_{{\text{i}}} \left( {\text{t}} \right) + {\text{v}}_{{\text{i}}} \left( {{\text{t}} + 1} \right)$$

**Fig. 2:**
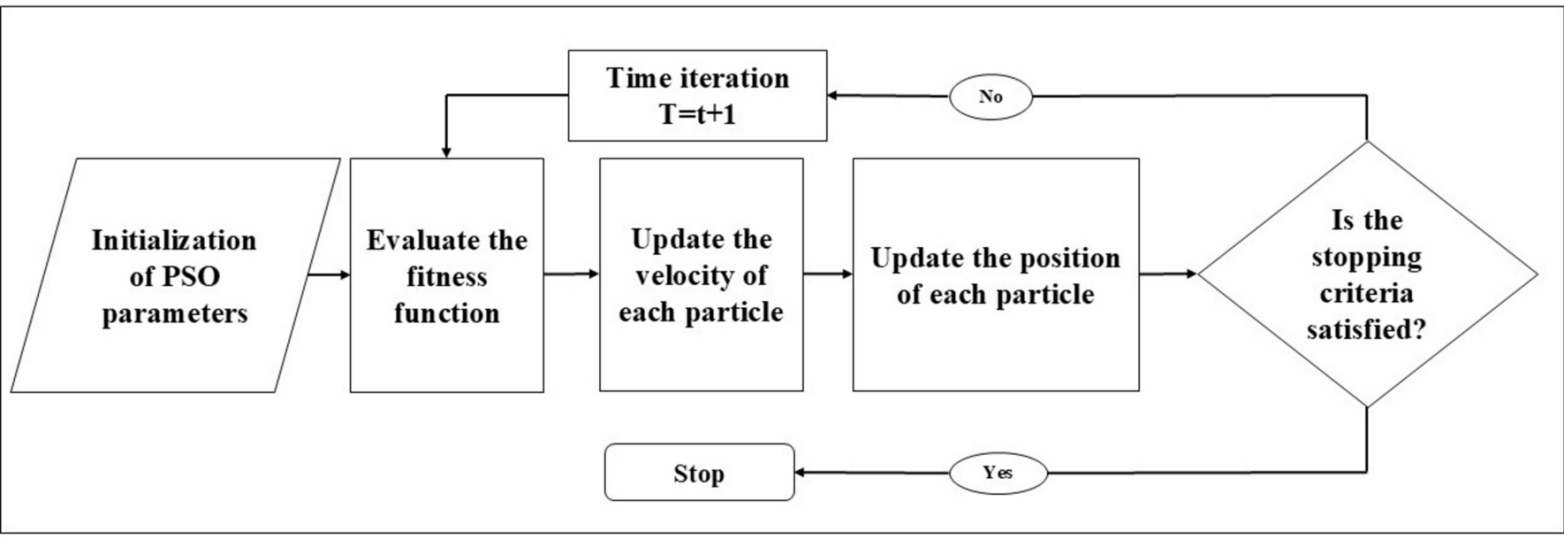
Basic working principle of particle swarm optimization (PSO).

Here, *vi*(*t*), vi(t) is the velocity of particle *i*i at time *t*t. *xi*(*t*), xi(t) is the position of particle *i*i at time *t*t. *pi*, pi is the best-known position of particle *i*i. *g*g is the global best position found by any particle. ωω is the inertia weight. *c*1, c1 and *c*2, c2 are cognitive and social coefficients. *r*1, r1 and *r*2, r2 are random numbers between 0 and 1. After that repeat the evaluation and update steps for a fixed number of iterations or until the change in the objective function is below a certain threshold. Figure 2 illustrates the flow chart of Particle Swarm Optimization (PSO).

### Discrete wavelet transform (DWT)

**Fig. 3:**
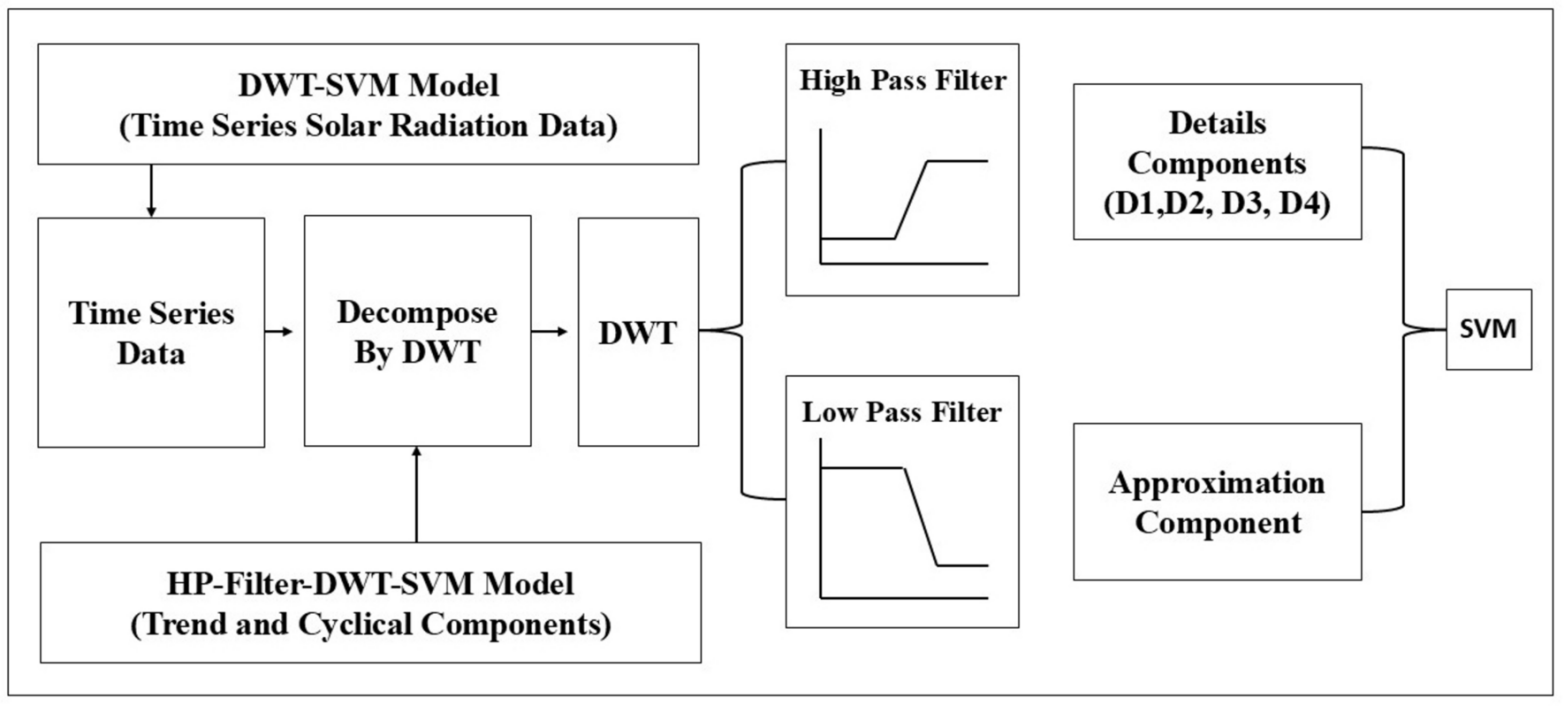
Working flow chart of DWT-based existing and proposed models.

**Fig. 4:**
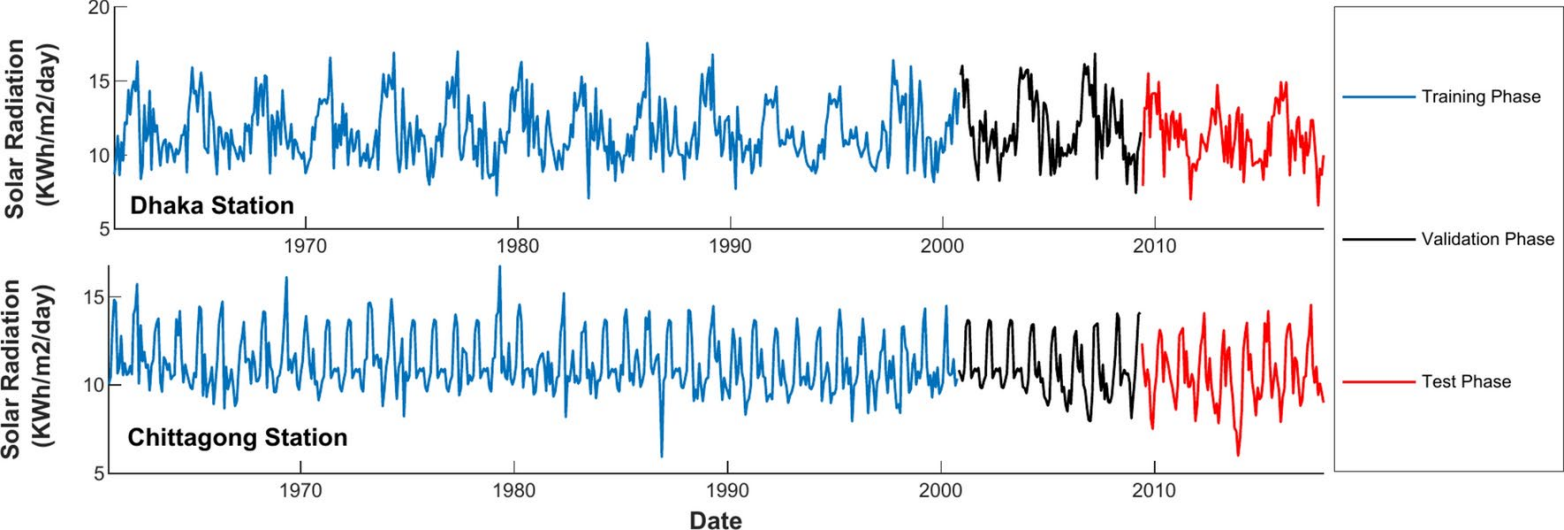
Time series plot of solar radiation.

Discrete Wavelet Transform (DWT) is a very powerful mathematical tool used in analyzing and processing signals, whereby the signal is broken down into a series of wavelets, small oscillations that exist for a finite duration^53^. It allows deconstruction at different resolutions of a signal; thus, it becomes useful where the time and frequency information of a signal is relevant in such tasks^54^. Essentially, DWT is a transformation method that maps signals from their original domain, most often time or space, to the wavelet domain^55^. The transformation involves passing a signal through a set of filters, usually a low-pass and a high-pass filter, and extracting different components of frequency at different scales illustrated in Fig. 3^32^. These coefficients represent the signal concerning these wavelets and can be used for further analysis or processing. One major advantage of the DWT is its flexibility to provide a multi-resolution analysis, capturing the low-frequency components that correspond to the trend of the signal and the high-frequency components that capture the details and abrupt changes^56^. It makes DWT especially suitable for applications such as signal compression and denoising, where the goal will be to save meaningful features while dispensing with other non-meaningful ones that make up large portions of a signal^57^. The mathematical background is given below (Fig. 4).

Assume x(t) is a continuous input time series where t $\epsilon$ [$\infty , -\infty$], a wavelet function $\Psi \left( \eta \right)$ that depends on *η*, can be defined as Eq. (13):

13 $$\Psi \left( \eta \right) = \, \Psi \, (\tau ,s) = s^{ - 1} \Psi \left( {\frac{t - \tau }{s}} \right)$$

where t represents time, τ is the timestep during which the window function is iterated, and s is the wavelet scale (0, ∞). The term Ψ(ƞ) must have zero mean and be localized in time as a Fourier space. Equation (13) calculates the level of similarity between the predictor data set and the wavelet function at various scales and translations. It produces a collection of wavelet coefficients that can be used to create a contour map.

In practice, input data is typically a set of discrete values (e.g., daily, monthly, seasonal). To deconstruct the signal, use the discrete wavelet transformation (DWT). DWT can select the translation and location settings for the input time series. The method then generates a set of discrete wavelet coefficients (DWCs) that represent the number of components required to reflect the input time series according to the mother wavelet’s equation, which is of the following form (Eq. 14)^32^.

14 $${\text{g}}_{{{\text{i}},{\text{j}}}} \left( {\text{t}} \right) \, = \frac{1}{{\sqrt {a_{0}^{i} } }}\left( {\frac{{t - jb_{0} a_{0}^{l} }}{{a_{0}^{i} }}} \right)$$

where i and j are integer values, and b0 and a0 are location parameters and the number of fine scale dilation steps supplied. In this investigation, the values of a0 and b0 were nominally set to 2 and 1 respectively, and the DWT process was able to pick the relevant scales and positions based on the powers of two (dyadic scales and translations), viz Eqs. (15) and (16):

15 $${\text{g}}_{{{\text{i}},{\text{j}}}} = 2^{{ - \frac{i}{2}}} g\left( {2^{ - i} h - j} \right)$$

16 $${\text{T}}_{{{\text{i}},{\text{j}}}} = 2^{{ - \frac{i}{2}}} \mathop \sum \limits_{h = 0}^{j - 1} g(2^{ - i} h - j)x_{h}$$

Note that T(i,j) is the wavelet coefficient for the discrete wavelet with a scale a = 2^i^ and location b = 2^i^ j, a finite time series, x_h_, h = 0,1,2,. . . , j − 1 and j is an integer power of 2, i.e., j = 2^i^. The inverse discrete transformation is then given by Eq. (17):

17 $${\text{X}}_{{\text{h}}} = \overline{T} + \mathop \sum \limits_{i = 1}^{l} \mathop \sum \limits_{j = 0}^{{2^{i - j} }} T_{i,j} 2^{{\frac{ - i}{2}}} g\left( {2^{ - i} - j} \right) = \overline{T} + \mathop \sum \limits_{i = 1}^{l} w_{i} \left( t \right)$$

where Wi(t) denotes the detail of the sub-series at level i = 1, 2,…, l, and T is referred to as the approximation sub-series at level i. For every predictor (input) variable in this study, four levels of detailed subseries and one level of approximation were produced. The mother wavelet can produce a set of wavelet coefficients that show how similar it is to the input dataset at particular scales as it is translated over the input signal. The generated wavelet coefficients, Wi(t) (i = 1, 2,…, l), can be used to analyze the various frequencies included in the predictor signal in terms of the large-scale (i.e., approximation) and small-scale (detailed) components. Notably, the residual term, T, represents the predictor dataset’s background information (long-term trends or low-frequency information) and so feeds critical information into the model for forecasting solar radiation. It is critical to note that the wavelet transformation procedure is a reliable method for pre-processing the model’s input data since it eliminates any potentially incorrect or parametric assumptions. Because of the precise information produced (i.e., W1(t), W2(t),…, Wl(t), relevant properties ‘hidden’ in the predictor dataset (e.g., periods, dependence, large amplitude perturbations, or jumps) are simply diagnosed and presented to the model.

Proper selection of the Daubechies wavelet as the mother wavelet in DWT is important for the accuracy of the signal decomposition process, which subsequently affects any model or analysis for which the DWT has been applied directly. The choice of ‘db’ wavelet influences how well DWT captures the features and patterns that exist within a signal. This study uses the Daubechies wavelet (db4) as the mother wavelet and selects a four-level decomposition based on relevant research^31,38^. Each level of the DWT represents different frequency bands, where lower levels capture high-frequency details and higher levels represent low-frequency, long-term trends. Solar radiation data at a monthly scale may contain both short-term variability (e.g., due to weather) and long-term variability related to seasonal change. Selecting four detail components D1, D2, D3, and D4 with the approximation A4, covers a range of frequencies to capture both high-frequency noise as well as low-frequency seasonal patterns.

### Hodrick–Prescott filter

The HP-Filter is among the most applied tools in time-series analysis, specifically for the disciplines of economics, finance, and environmental sciences. Its principal objective is to decompose any time series into two alternative components: the smooth long-term trend and short-term fluctuations or components that are, explicitly, cyclical. The separation makes it easy for any analyst to understand and isolate the underlying patterns in the data, and this easily identifies trends, cyclical movements, and anomalies. The HP filter was developed specifically in response to a common deficiency in the analysis of time series data, where the dataset showed long-term trends mixed up with short-term fluctuations. In most cases, the raw data may just be too noisy or volatile to make meaningful interpretations. The trend term from Hp-Filter represents the long-term movement that the data revolves around. This captures well-sustained movements or the “structural” part of the data that gradually evolves. This could involve the general economic growth path in macroeconomics or the general underlying trend in stock prices in finance. A Cyclical Component from Hp-Filter picks out the short-term deviations from the trend. Such can be thought of as business cycles or seasonal effects, among other types of noise. Suppose that y_t_ is a time series, such that ${\tau }_{t}$ is the trend component and ${c}_{t}$ the cyclical component, so that y_t_ = ${\tau }_{t}$ + ${c}_{t}$ .Then choosing a suitably positive value for $\lambda$ Eq. (18) (1) may be used to deduce the trend component.

18 $$\mathop {\min }\limits_{\tau } \left( {\mathop \sum \limits_{t = 1}^{T} (y_{t} - \tau_{t} )^{2} + \lambda \mathop \sum \limits_{t = 2}^{T - 1} \left[ {\left( {\tau_{t + 1} - \tau_{t} } \right) - \left( {\tau_{t} - \tau_{t - 1} } \right)} \right]^{2} } \right)$$

The first term of this equation includes the sum of the squared deviations d_t_ = y_t_ − ${\tau }_{t}$ , which very much penalizes the cyclical. The second term is a coefficient times the sum of the squares of the second difference of the trend component. This term penalizes fluctuations in the growth rate of the trend, where larger values of $\lambda$ correspond to a higher penalty. Hodrick and Prescott suggest using 1600 as the value for λ for quarterly data. In general, following Ravn and Uhlig, 2002, λ should scale with the fourth power of the ratio of observation frequencies. For annual data, λ should be 6.25, and for monthly data, it should be 129,600. In practice, though, λ = 100 is used with yearly data, and λ = 14,400 with monthly data^58^. In this study, the value of λ in the HP-Filter was adopted as 14,400, a commonly accepted parameter when analyzing monthly data. The smoothing parameter λ in the HP filter regularizes the sensitivity of the trend component to short-term fluctuations. One of the standard values using 14,400 for monthly data provides a good trade-off between the smoothness of the trend and capturing meaningful seasonal or cyclical patterns^59^.

## Research methodology

The overall process is divided into several sections: reviewing existing research, identifying limitations, selecting a study area and data preparation, developing a unique model to address the limitations, and comparing the proposed model’s performance to existing methodologies. A literature analysis provided some background information on several conventional and hybrid strategies for predicting solar radiation. The primary insights from previous research were noisy data handling and error minimization. To address the shortcomings noted with existing methodologies in this regard, this study produced three modeling approaches: one traditional method, one hybrid approach, and one proposed novel approach. The proposed approach would address the shortcomings of conventional and existing hybrid techniques. The first two important sections, such as the literature review and finding limitations, are discussed in the introduction section.

## Research location and data preparation

**Fig. 5:**
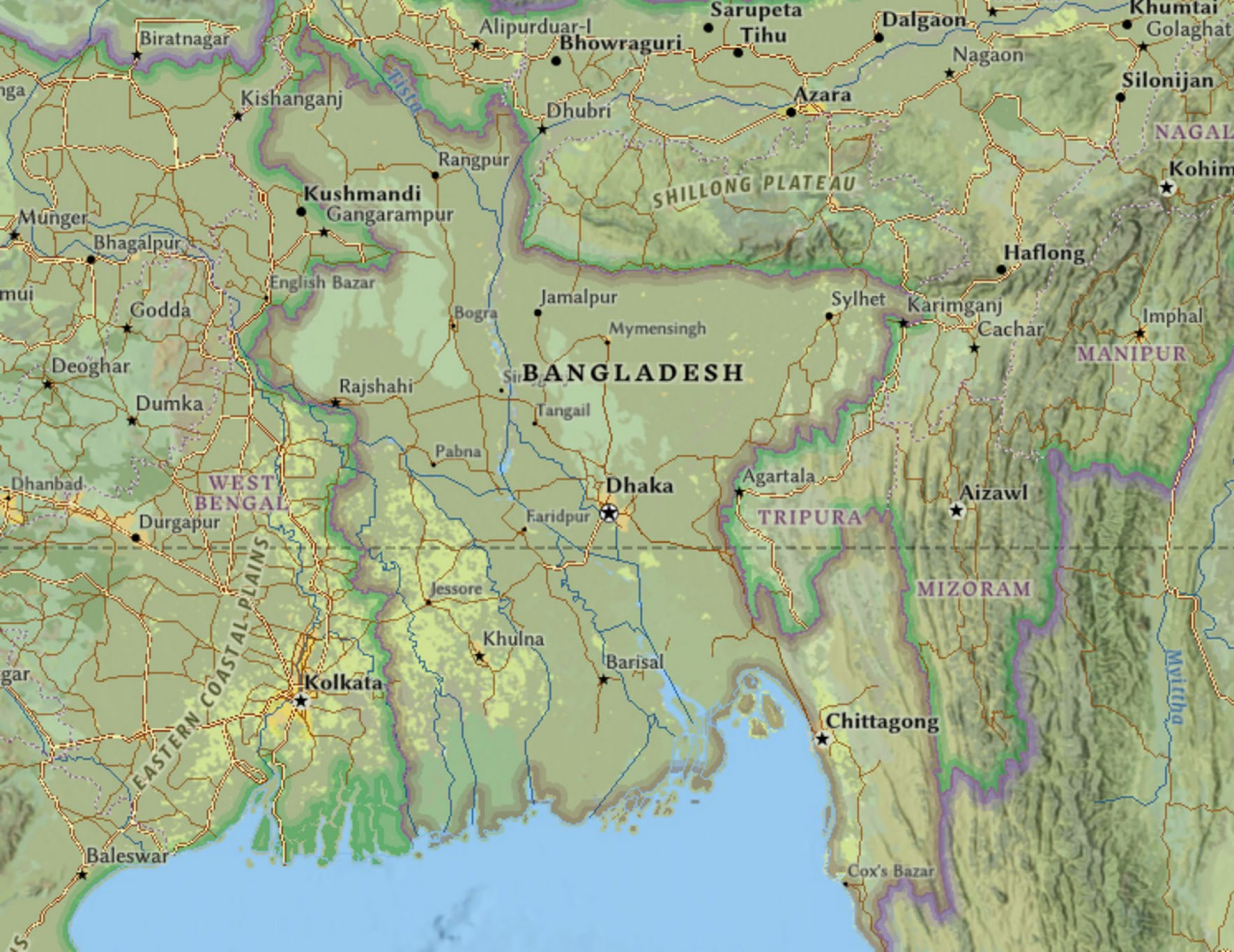
Study area map.

The two different locations chosen for this research are Dhaka (23° 46′ 37.8336′′ N, 90° 23′ 58.0272′′ E) and Chittagong (22.22° N, 91.47° E). These cities have been selected strategically because they are of different latitudes and longitudes, hence increasing the representativeness of the study. The locations are shown in Fig. 5, created using the 'ArcGIS Online Map Viewer’ (https://www.arcgis.com/home/webmap/viewer.html). Solar radiation data from January 1961 to December 2017 for both locations were collected from the Bangladesh Meteorological Department (BMD). These 57 years of vast historical monthly data thus enhance models of solar radiation prediction by catching long-term trends, seasonal variations, and anomalies in them, hence making the predictions more accurate and stronger^12^. The solar data were arranged by averaging morning, afternoon, and evening solar radiation. Averaging solar radiation measures taken at different times of the day smooth out variability due to changes in the sun’s position and atmospheric conditions. This would provide a more stable and representative measure of solar energy received throughout the day. This will reduce the impact of anomalies on any studies connected with energy, agriculture, and climate, and it will provide more accurate data. Averaged values also facilitate consistent comparisons and improve the calibration and validation of predictive models. The data was divided into three phases: the training phase from January 1961 to November 2000, the validation phase from December 2000 to June 2009, and the testing phase from July 2009 to December 2017. The division described in Fig. 4 allows for comprehensive model development and evaluation to enhance the accuracy of the predictions and make them more robust.

### Model development

#### Lag-time based single traditional SVM model: SVM

In this research, the single traditional SVM model was developed using the lag time of solar radiation as a key feature. By lag time, it refers to how the current time step may be correlated with any previous time steps. For example, in predicting the monthly radiation, the current month’s radiation may be impacted by the previous months’ radiation levels. By introducing this lag, the SVM model can capture temporal dependencies, thereby allowing it to make accurate predictions of future values. The basic idea is that solar radiation patterns are periodic, and considering past data will help the model get a better understanding and forecast of the values. This approach uses the fact that solar radiation is not a random process but follows seasonal trends and patterns that are influenced by various atmospheric factors. This is particularly appropriate since the SVM model is very robust and efficient in handling nonlinear relationships. In this case, the introduction of the lag time as a feature.

enables the model to efficiently map the relationship between the past and current radiation levels, thus improving its prediction accuracy^60^. This technique becomes of further value when used in areas related to renewable energy forecasting. In this area, understanding the patterns of solar radiation forms the underpinning for the optimization of energy production and grid management.

The methods of lag time have been applied in the past for the prediction of solar radiation to take into consideration the temporal relationship between the current and past values^61–63^. The significant lag to be used for modeling is selected by using the Partial Autocorrelation Function plot. It shows the direct relationship between a time series and its lags, removing the contributions of the intermediate lags, thus showing which lag time has a great influence on the current time step. In the PACF plot, one can identify which time lags should be included in the model to capture the influential lags for increasing the predictive ability. The PACF plots for various stations are also given in Fig. 10, which shows how the significant lags differ at different locations. Such an approach would ensure that all the principal temporal patterns are captured by the model and result in better solar radiation predictions for decision-making in renewable energy management. Figure 10 shows important lags at 1, 2, and 3; therefore, these specific lags are significantly correlated with the current value of a time series on solar radiation. This tells that the past values observed at these time points hold some useful information that may be predictive of the current level of solar radiation. Identifying such significant lags underlines the importance of temporal dependencies within time-series data, more so in the context of solar radiation prediction.

This paper uses the data from the preceding time points t − 1, t − 2 and t − 3 as input features to a machine learning model that is then trained to estimate the solar radiation level at time t. The connection between the current value of solar radiation and each of its previous values can be formalized with the help of the following equation Eq. (19):

19 $${\text{y}}_{{\text{t}}} = {\text{ f}}({\text{y}}_{{{\text{t}} - {1}}} ,{\text{ y}}_{{{\text{t}} - {1}}} ,{\text{ y}}_{{{\text{t}} - {1}}} ) \,+ \in$$

where, y_t_ is the value of the time series at the time, 't', 'f' is the function learned by the machine learning model, y_t−1,_ , y_t−1_, and y_t−1_ are the lagged values of the series; '$\in$' is an error term that refers to any kind of noise or pure randomness that cannot be predicted by the model.

#### Discrete wavelet transform (DWT) based SVM model: DWT-SVM

**Fig. 6:**
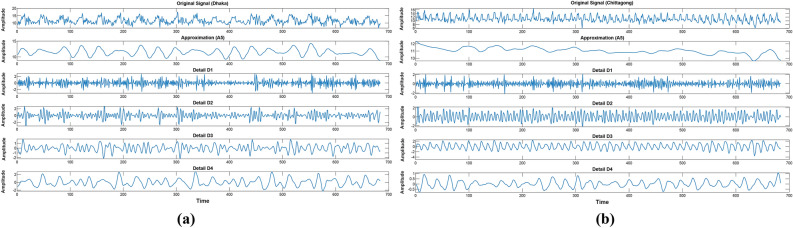
DWT decomposed solar radiation data (**a**) Dhaka station and (**b**) Chittagong station.

In this section, a model trained with a Discrete Wavelet Transform will be applied. In this step, details and approximation components of solar radiation data obtained by DWT processing will be used as input to the model. The DWT is an advanced method of time–frequency analysis; it is used for the decomposition of the time series into a few frequency components in which every level shows different details and approximations. On the other hand, applying DWT to solar radiation data breaks down the original time series into a set of sub-signals that may be viewed as capturing some features of the data at different scales. The approximation component retains the trend of data with low-frequency characteristics, while the detailed components describe high-frequency variations, reflecting more transient or abrupt changes in the data. These decomposed signals, with their more sophisticated and detailed representation of the original time series, enable the model to learn from the general trends as well as finer details in the data. The components of the DWT-SVM model can help a great deal in effectively capturing both long- and short-term variations in solar radiation, therefore improving predictive performance. Figure 6 shows the DWT decomposed signal and Fig. 3 depicts the integration process between DWT and SVM. One can visibly notice the representation using wavelet-transformed data during the model training phase.

Integration of DWT with SVM provides a strong method for time series prediction, especially for complex and non-stationary data like solar radiation. In this respect, DWT makes the model work on multi-resolution analysis by focusing on relevant features at different granularities, while SVM is applied as a strong framework to capture the nonlinear relationship between inputs and the target variable. This interaction between DWT and SVM makes the model very powerful in predicting solar radiation with a high degree of accuracy, thus rendering it a much-needed tool in renewable energy forecasting and related fields.

#### Hodrick–Prescott filter-based DWT-SVM model: HP-filter-DWT-SVM

**Fig. 7:**
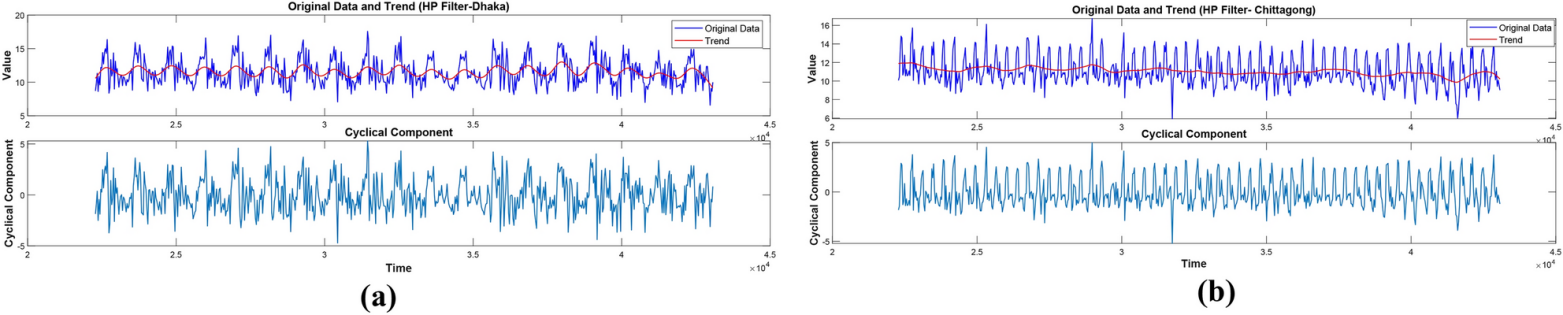
HP-Filter decomposed solar radiation (**a**) Dhaka station and (**b**) Chittagong station.

This section presents the proposed methods for constructing a hybrid model for enhanced solar radiation prediction using the Hodrick-Prescott Filter, Discrete Wavelet Transforms, and Support Vector Machine. This will start with the application of the HP filter, one of the most prevalent tools in time series analysis. It efficiently decomposes data into cyclic and trend components. The HP filter extracts the underlying patterns in the data, separating the long-term trends from the short-term fluctuations. It cleans up the signal for further analysis. The trend and cyclic components of the HP-Filter for both samples are illustrated in Fig. 7.

**Fig. 8:**
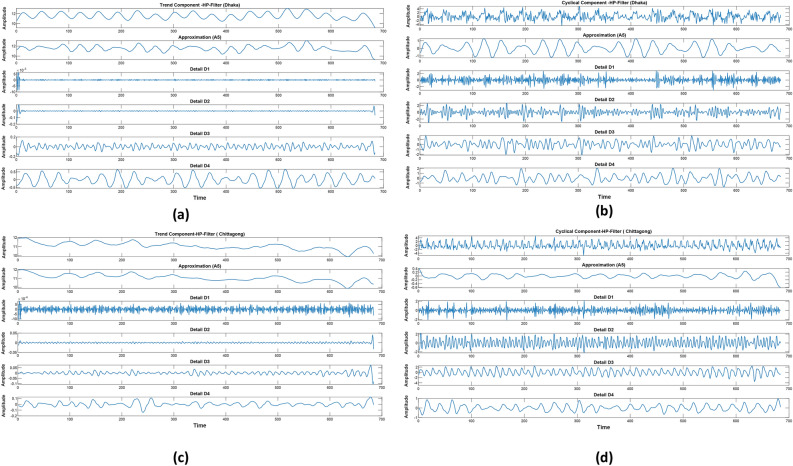
Decomposition of the trend component (Dhaka (**a**), Chittagong (**c**)), decomposition of the cyclic components (Dhaka (**b**), Chittagong (**d)**).

**Fig. 9:**
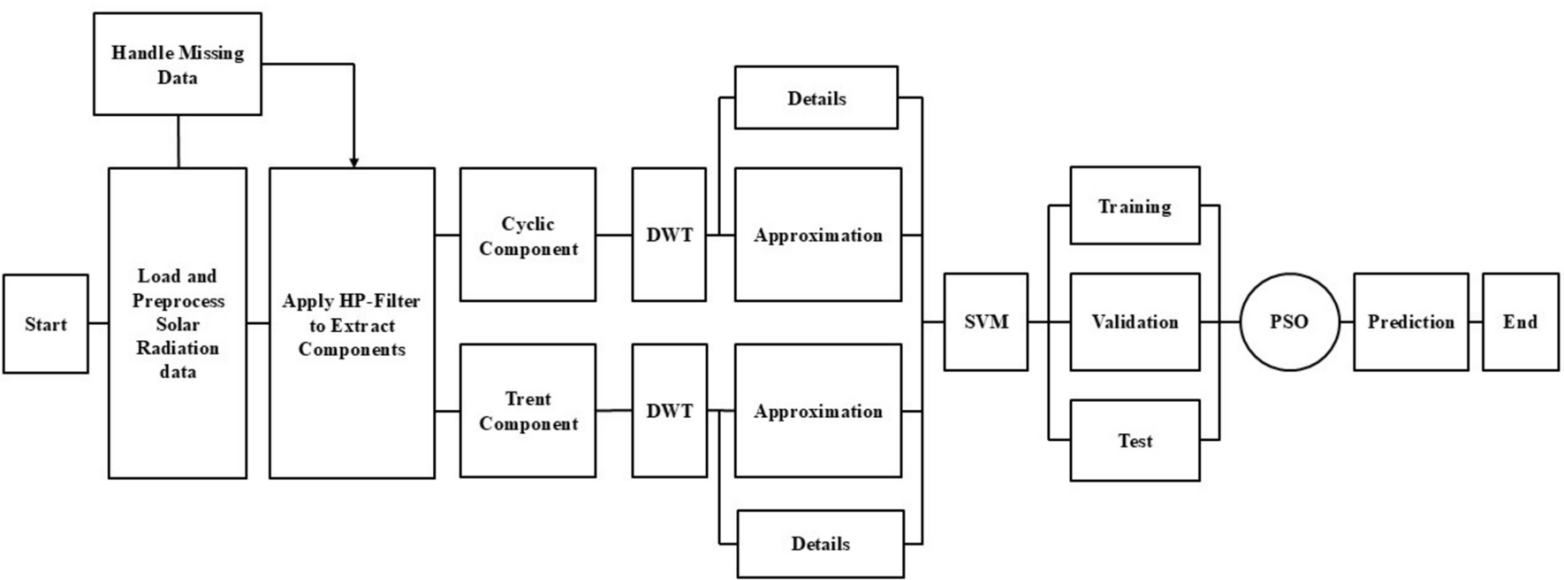
Overall flowchart of the proposed methodology.

After the HP-Filter has successfully split the data into its trend and cyclic components, a more detailed analysis will be performed on each of these components using the DWT. The DWT performs a multilevel decomposition of data, decomposing trend and cyclic components into finer sub-signals. Figure 8 depicts the DWT decomposed signal for the trend and cyclic components. These will include approximation components, which capture broad, low-frequency trends, and detail components, focusing on high-frequency variations. These decomposed signals are fed into the SVM so that it can learn from a comprehensive set of features that represent various scales of data. The methodology fully exploits the advantages of both the HP-Filter and DWT by integrating them for noise removal, emphasizing the main patterns of information, and capturing information at different time scales in detail. The overall proposed methodology is illustrated in Fig. 9.

#### Statistical evaluation

**Fig. 10:**
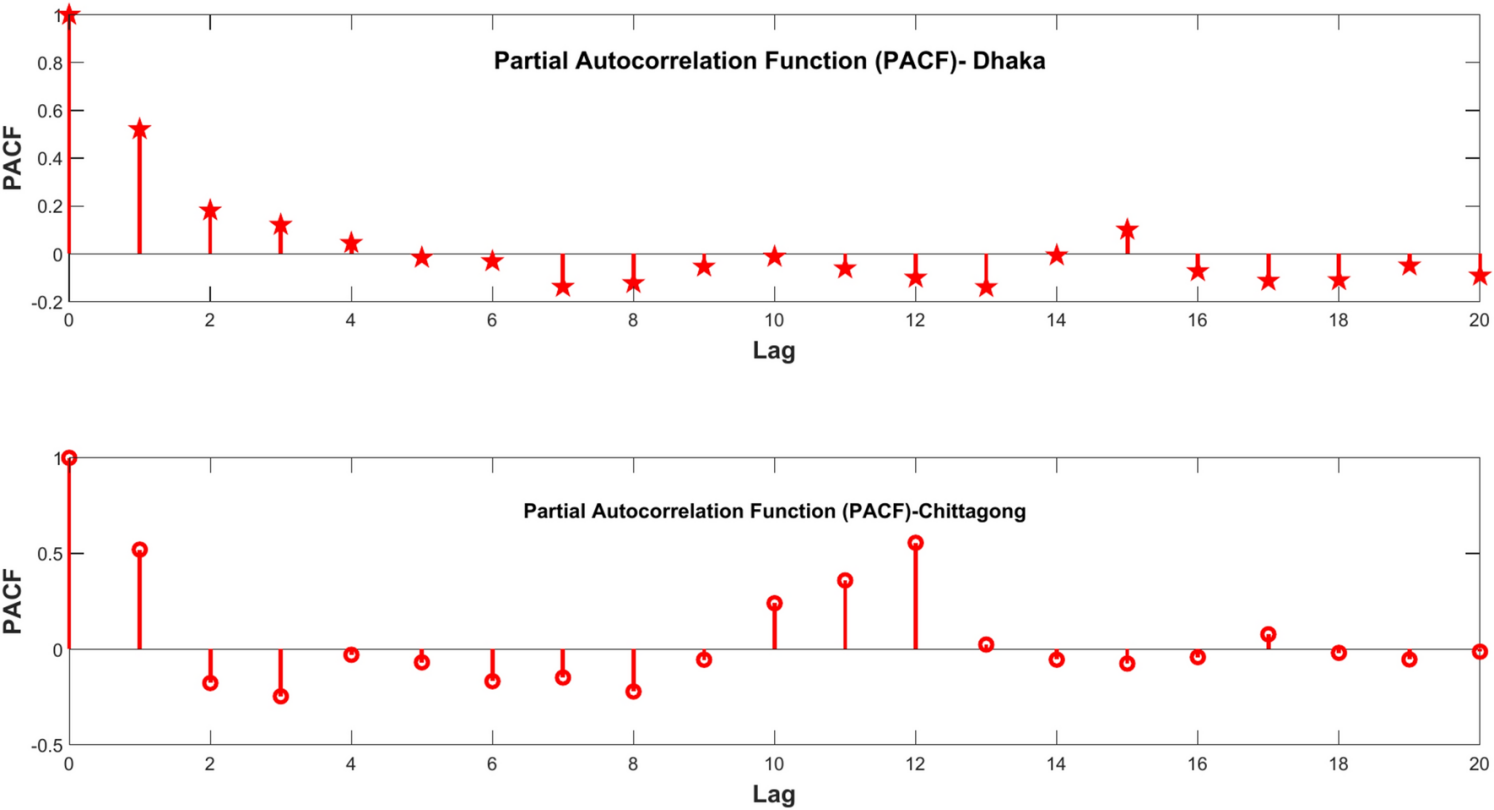
PACF plot of solar radiation.

Accuracy and efficiency may be quantified using several key statistical metrics, all of which provide different insights into predictive model output performances. The principal metrics used in this research are the mean squared error, root mean squared error, mean absolute error, mean absolute percentage error, and coefficient of determination. These metrics were chosen because of their common application and, thus, the ease of comparison that exists in many existing research pieces. MSE calculates the average of the squared differences between predicted and actual values; therefore, it gives greater weight to larger errors (Eq. 20). Its square root, RMSE, is a measure of error in the original units of data and hence more interpretable (Eq. 21). For MAE, the average absolute differences between the predicted and actual values provide a simple measure of error without squaring, helping to maintain the average prediction error directly interpretable (Eq. 22). MAPE provides the average of the absolute percentage error to return a relative measure of error in terms of percentage values, which makes the errors more comparative across different scales (Eq. 23). R2 returns the proportion of variance of the dependent variable around the mean explained by the independent variables, and it shows how well values are predicted with the proposed model (Eq. 24). This set of metrics provides a complete overview of model performance and can ensure good understandings of predictive accuracy. Table 2 shows the equation of the all-performance matrix (Fig. 10).

**Table 2:** Equation of the evaluation matrix.

Evaluation matrix	Equation	Description
Mean square error (MSE)	${\text{MSE}} = \left( \frac{1}{n} \right)\sum (y_{i} - \overline{y})^{2}$ (20)	Where n is the number of data points, y_i_ is the ith actual value, ȳ is the mean of the actual values
Root mean square error (RMSE)	${\text{RMSE}} = \sqrt {\left( {{ }\frac{1}{n}{ }} \right){ }\sum (y_{i} { } - { }\overline{y})^{2} }$ (21)	Where n is the number of data points, y_i_ is the ith actual value, ȳ is the mean of the actual values
Mean absolute error (MAE)	${\text{MAE}} = \left( {{ }\frac{1}{n}{ }} \right) \sum (y_{i} { } - { }\overline{y})^{2}$(22)	Where n is the number of data points, y_i_ is the i^th^ actual value, ȳ is the mean of the actual values
Mean absolute percentage error (MAPE)	${\text{MAPE}} = \frac{1}{n}\sum\nolimits_{i = 1}^{i = n} {\left| {\frac{yi - xi}{{yi}}} \right|} \times 100\%$ (23)	yi = Actual value, xi = predicted value, n = number of possible data sets
Coefficient of determination (R^2^)	$R^{2} = 1 - \left( {\frac{{\sum \left( {y_{i} - \overline{y} } \right)^{2} }}{{\sum \left( {y_{i} - \hat{y} } \right)^{2} }}} \right)$(24)	Where y_i_ is the i^th^ actual value, ȳ is the mean of the actual values, ŷ is the predicted value

## Result and discussion

This research proposes a novel hybrid model for predicting solar radiation that combines the Discrete Wavelet Transform, Hodrick-Prescott Filter, and Support Vector Machine. It aims to improve prediction accuracy by leveraging the inherent capabilities of these approaches in signal decomposition, trend smoothing, and machine learning. In this study, the proposed approach was implemented and compared to previously existing methods and models based on DWT and lag time analysis to demonstrate its efficiency, as found in studies^32,60–63^.

The accuracy of any prediction model depends on the proper choice of input parameters. The literature discussed several researchers who have tried to get maximum accuracy by using a large number of predictive input elements. This research is unique in the sense that it only uses historical solar radiation data from 1961 to 2017 for the development of models. In the single traditional SVM model development section, the selection process has been briefly outlined before for the input features with the help of the Partial Autocorrelation Function plot. As presented in Study^32^, the hybrid DWT-SVM model will use both the detail and approximation coefficients obtained from the DWT analysis for feeding purposes into the SVM model. At this point, the proposed method will further enhance the predictive performance of the DWT-SVM approach by using a Hodrick-Prescott Filter. In the proposed hybrid approach, the HP-Filter is applied before the DWT decomposition. This is followed by the DWT of the HP-Filter components to obtain a more fine-tuned and accurate prediction model. To further assess the developed approach’s robustness and reliability, the analysis is extended and applied to estimating the monthly mean daily global solar radiation. The results are presented and discussed in some detail for a comprehensive evaluation of the model’s performance in the distinct sub-sections below.

### Prediction of monthly solar radiation

To predict the monthly global solar radiation, the dataset is divided into 70% training to ensure that algorithms have learned from a significant part, 15% validation for fine-tuning optimal SVM hyperparameters using the Particle Swarm Optimization algorithm, and another 15% for testing the predictive capability of the trained models. This was an important step in the model performance optimization before it was applied to the final 15% reserved for testing. In this study, three different models were considered: traditional SVM, hybrid DWT-SVM, and proposed hybrid HP-Filter-DWT-SVM. For each model, the forecast monthly global solar radiation data were compared to the measured data under a set of very well-known indicators. These included the Mean Squared Error, Root Mean Squared Error, Mean Absolute Error, Mean Absolute Percentage Error, and Coefficient of Determination.

The results of this analysis are reported in Table 3, with a view to the test phase. As far as these indicators are concerned, in general, the goodness of fit is explained by the statistical indicators: the lower the MAE, MAPE, RMSE, and MSE, the greater the precision of the predictions, meaning that the model is very good at estimating solar radiation. Conversely, high R2 values indicate a stronger linear relationship between predicted and observed values; an R2 value approaching + 1 indicates an almost perfect fit. This rigorous assessment thus underlines the effectiveness of the proposed methodologies in estimating the monthly global solar radiation.

**Table 3:** Model performances of the test sample.

Location	Model type	Performance matrix	Input feature	MSE	RMSE	MAE	MAPE	R^2^
Dhaka station	Traditional model	SVM-1	t − 1	2.73	1.65	1.33	0.1261	0.39
		SVM-2	t − 1, t − 2	2.54	1.59	1.3	0.1218	0.38
		SVM-3	t − 1, t − 2, t − 3	2.64	1.62	1.28	0.1212	0.4
	Hybrid model	DWT-SVM	Details and approximation	0.01	0.1	0.08	0.0081	0.91
	Proposed model	HP-Filter-DWT-SVM	Details and approximation	0.0061	0.078	0.063	0.0059	0.93
Chittagong station	Traditional model	SVM-1	t − 1	2.67	1.63	1.32	13.58	0.35
		SVM-2	t − 1, t − 2	2.3	1.51	1.22	12.44	0.47
		SVM-3	t − 1, t − 2, t − 3	1.9	1.38	1.11	11.59	0.44
	Hybrid model	DWT-SVM	Details and approximation	0.01	0.1	0.07	0.0077	0.95
	Proposed model	HP-Filter-DWT-SVM	Details and approximation	0.0043	0.06	0.05	0.0052	0.98

The three SVMs use time-lagged inputs to predict: SVM-1 uses t-1, SVM-2 uses t-1, t-2, and SVM-3 uses t − 1, t − 2, t − 3. These models also show the highest error rates in all metrics, with the MSE values ranging from 2.54 to 2.73 for the Dhaka station and from 1.9 to 2.67 for the case of the Chittagong station. RMSE values provide some idea about the average magnitude of the prediction errors; hence, these values are very high, too, falling between 1.59 and 1.65 for Dhaka, while those of Chittagong range from 1.38 to 1.63. Similarly, the MAE, which is the average absolute difference between predicted and actual values, also ranges from 1.28 to 1.33 at Dhaka and 1.11 to 1.32 at Chittagong. As shown, SVM models give the highest MAPE values, too, 12.12–13.58%, thus indicating a high percentage error in predictions. The values of R^2^ for these models range from 0.35 to 0.44, showing a weak correlation between predicted and observed values.

The DWT-SVM approach, which includes details and approximations of the Discrete Wavelet Transform, shows some noticeable advantages over traditional SVM models. The DWT-SVM model reduces the error metrics for both stations significantly. For instance, at the station in Dhaka, MSE drops to 0.01, RMSE to 0.1, MAE to 0.08, and MAPE to 0.81%. The value of R^2^ has increased significantly to 0.91, which is indicative of the strong linear association between the predicted and actual values. For the Chittagong station, the MSE was equal to 0.01, RMSE = 0.1, MAE = 0.07, and MAPE = 0.77%, with R^2^ = 0.95 for the DWT-SVM model. These improvements could be understood to mean that the DWT-SVM model is better at handling the complexities of the data, likely because it is capable of decomposing the signal into different frequency components that would enhance the model’s ability to capture relevant features.

**Fig. 11:**
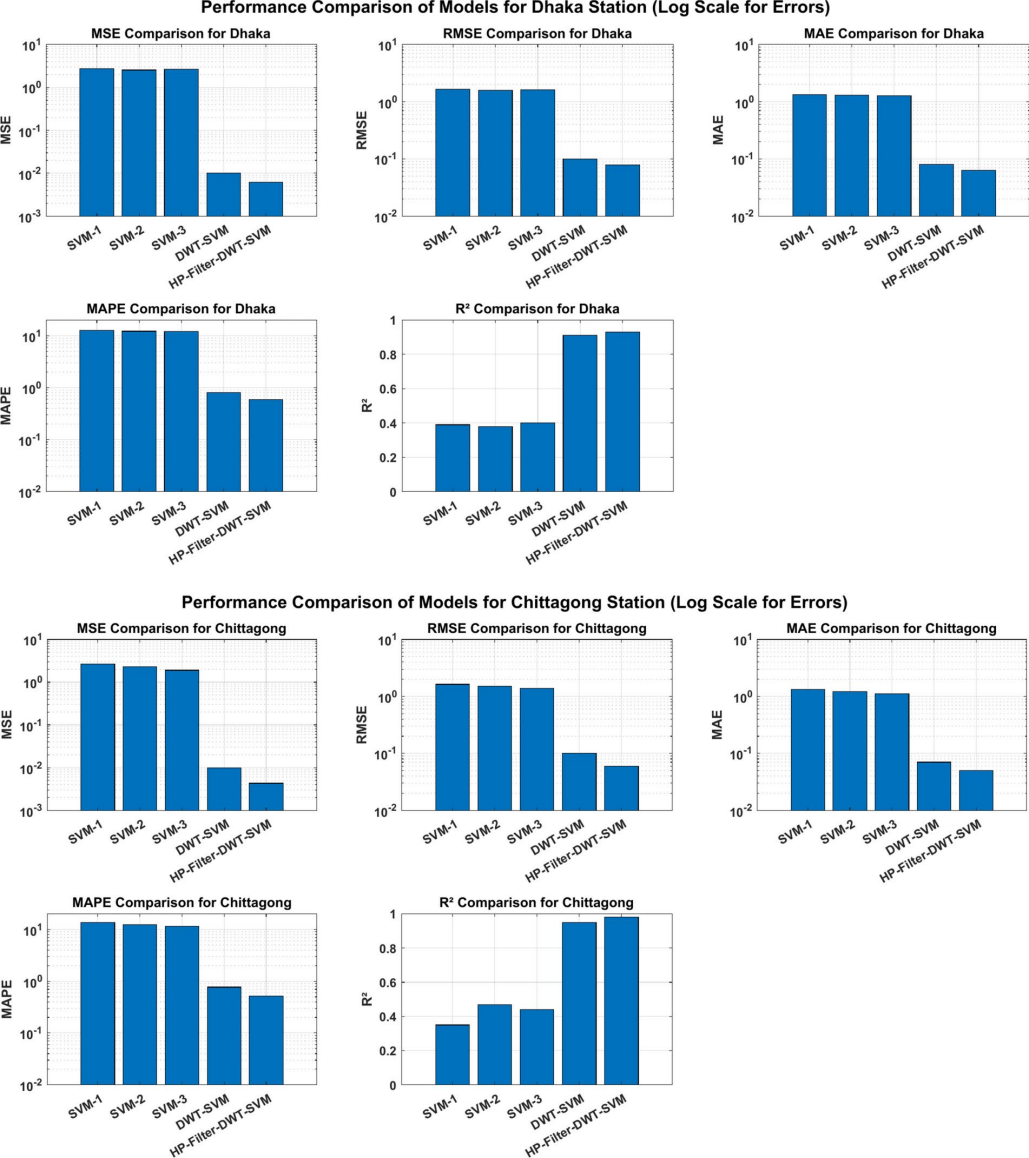
Visual comparison of the performance metrics by the proposed method with the traditional SVM and hybrid DWT-SVM approaches.

**Fig. 12:**
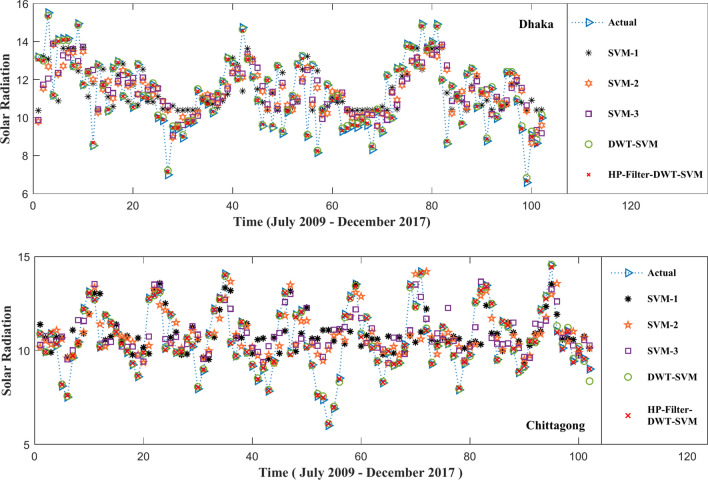
Actual versus predicted outcomes of test sample.

In this regard, the HP-Filter-DWT-SVM approach further refines the input data with an HP- Filter before using DWT and SVM and then outperforms both traditional SVM and hybrid DWT-SVM models concerning of metrics. The HP-Filter- DWT-SVM model presents results with an MSE of 0.0061, RMSE of 0.078, MAE of 0.063, and MAPE of 0.59% at the Dhaka station. A strong relationship, indicated by the R^2^ of 0.93, would be an indicator that the model is very good at capturing essential patterns in the data. The performance is even significantly better at the Chittagong station, where the MSE of the implemented model has dropped to 0.0043, the RMSE to 0.06, the MAE to 0.05, and the MAPE to 0.52%, with an R^2^ of 0.98. These findings represent the robustness of the HP-Filter-DWT-SVM approach, in which the prediction error is reduced and the explained variance is increased. Therefore, it is the most precise and reliable model for predictive tasks in this paper. Figure 11 graphically illustrate all of the metrics for different methods, and it is clear that the proposed technique outperforms both the traditional SVM and the hybrid DWT-SVM models. The actual vs. predicted plot is shown in Fig. 12.

To optimize the model of the Support Vector Machine for predicting monthly global solar radiation, it is necessary to find the optimal hyperparameters. Especially finding a best-fit kernel function along with the optimal values for the regularization parameters C and Gamma. In this research, the PSO algorithm is used to find out these optimal hyperparameters. After running the PSO, the linear kernel function always turned out to be the best choice for all cases, including the datasets from Dhaka and Chittagong. Moreover, it was determined by PSO that the optimal value for the regularization parameter c was 10, and gamma 1 for both Dhaka and Chittagong.

### Comparison with the lag-time (SVM) and wavelet transform (DWT-SVM) based model:

The HP-Filter-DWT-SVM model significantly improves predictive accuracy over the traditional SVM models and the hybrid DWT-SVM method. In the Dhaka station, this model can reduce MSE by about 99.76% compared with SVM models and 39% concerning the DWT-SVM model. About 95.09% relative to the SVM models, and about 22% relative to the DWT-SVM model. MAE is reduced by 95.08% relative to the SVM models and by 21% relative to the DWT-SVM model, while the MAPE around 95.66% decreases relative to the SVM model and 27% in the DWT-SVM model. The value of the coefficient of determination (R^2^), of the HP-Filter-DWT-SVM model further increases by 49% over the SVM models and by 4.40% over the DWT-SVM model, which suggests a higher strength of the relationship between predicted and observed values.

**Fig. 13:**
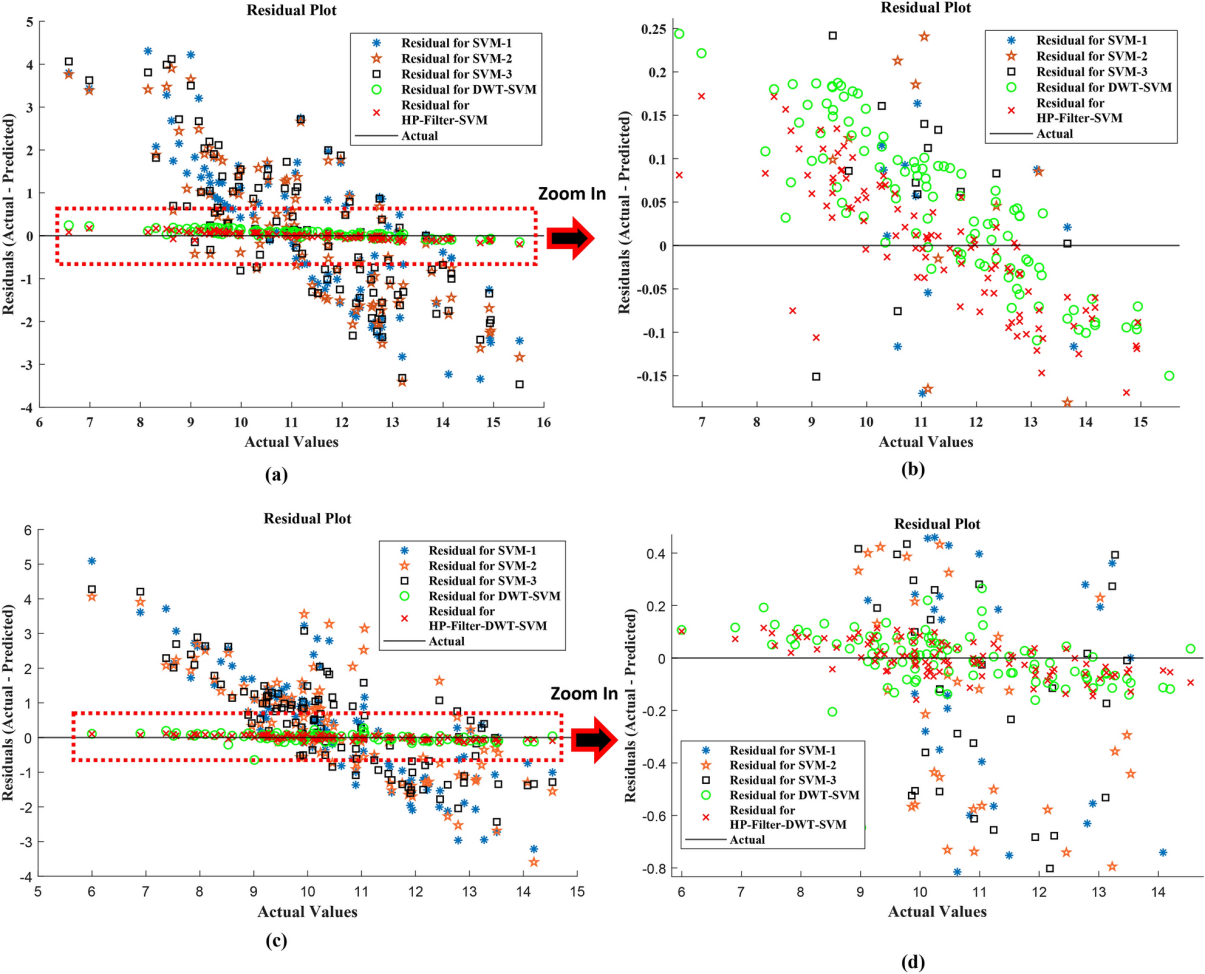
Residual plot of the actual versus predicted test samples. (**a**) Dhaka station; (**b**) zoom in plot of Dhaka station; (**c**) Chittagong station; (**d**) zoom in plot of Chittagong station.

For the station at Chittagong, the inferences are even more marked. Concerning the SVM models, the HP-Filter-DWT-SVM model decreases MSE for that station by 99.77%, and relative to the DWT-SVM model, it decreases it by 57.0%. Compared to the SVM models, the RMSE is decreased by 95.65% whereas, when compared to the DWT-SVM model, it is decreased by 40%. The MAE is reduced by 95.49% when compared to the SVM models, by 29% when compared to the DWT-SVM model, and the MAPE lowered by 95.75% concerning the SVM models and by 32% concerning the DWT-SVM model. The R^2^ value is increased by 54% compared to the SVM models and by 3.16% over the DWT-SVM model, further pointing out the strength of the HP-Filter-DWT-SVM approach. These findings indicate that the HP-Filter-DWT-SVM model significantly reduces the prediction error along with an increase in the explained variation and proves to be the most precise and reliable method for predictive tasks in this study. It can be seen from Fig. 11, where the comparison between the actual and predicted results for all the models is illustrated, and it can also be seen from Fig. 13, with a residual plot that includes a zooming section for better vision.

### Comparison with the existing research

Comparing the proposed model with existing models, it becomes quite evident that the hybrid HP-Filter-DWT-SVM model is far better than the existing models, as detailed in Table 1 and discussed in the introduction. The key innovation in this model lies in the application of the HP- Filter before the Discrete Wavelet Transform (DWT). It is this preprocessing step that aids in the effective removal of low-frequency noise and ensures that DWT can capture relevant signal characteristics accurately. It has been possible to improve the accuracy over existing techniques and also justify the effectiveness of incorporating the HP- Filter in the preprocessing stage by reducing the prediction errors considerably. Further improvement underpins the potential of the HP-Filter-DWT-SVM model to offer more reliable and precise predictions, hence becoming an important tool in the domain of solar radiation forecasting.

**Fig. 14:**
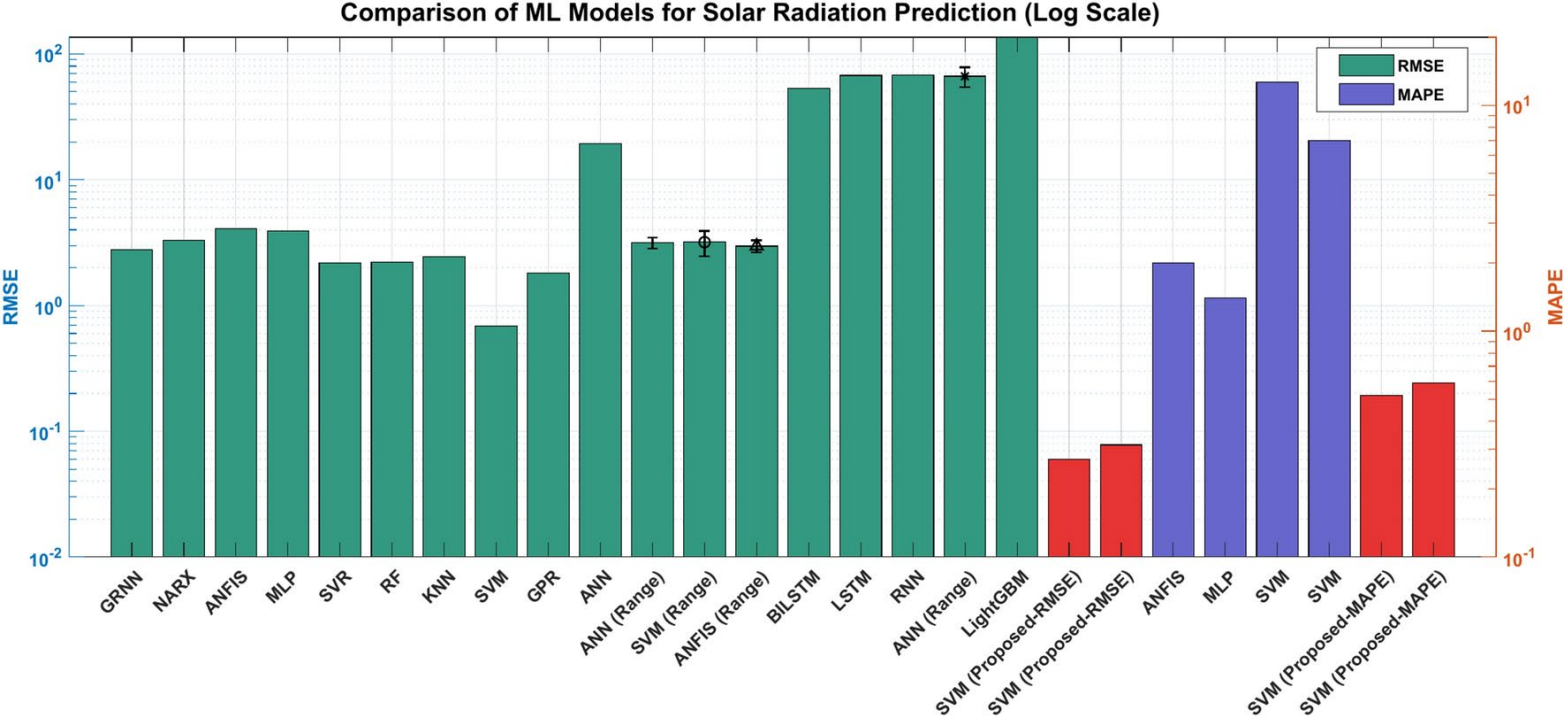
performance comparison of proposed model against existing machine learning and deep learning approaches.

A comparison plot of previous studies and the outcomes of the proposed approach based on RMSE and MAPE is shown in Fig. 14, which demonstrates the proposed technique’s significant advantage over several machine learning and deep learning models. The comparison underlines the fact that the application of the HP filter before the implementation of DWT significantly enhances the ability of the SVM model for the analysis and forecast of solar radiation. This step of preprocessing with the HP filter helps in distinguishing the trend from the cyclic components so that the model can concentrate on the more intricate patterns within the data, which are crucial for making more accurate predictions. The result is that this joint processing using the HP filter and the DWT yields an effective capture of complex seasonal and time-varying features in solar radiation, hence enhancing the predictive capability of the model compared to the use of DWT only with the Support Vector Machine.

### Sensitivity analysis

Sensitivity analysis, therefore, plays an important role in modeling and prediction to see the impacts of changes in model parameters on the model outcomes. The wavelet-based machine learning model previously demonstrated that the choice of mother wavelet has a considerable impact on prediction accuracy^43^. Sensitivity analysis is required to prove the robustness and reliability of the model; it systematically changes certain factors such as the selection of the mother wavelet or the decomposition level to find which relationships between parameters most influence the prediction result. That will not only help choose the best wavelet for any dataset given but also be very informative about model limitations and hence possible weaknesses. It also helps the researcher in model fine-tuning for better performance, enables interpretability of models, and sets a basis for comparison of the model’s stability across configurations. This insight into these aspects is very helpful in securing the effectiveness of the model under changing conditions, which on the other hand, becomes very important to consider in applications like studies dealing with solar radiation, where high accuracy and precision become vital elements of concern.

#### Chittagong station

During the development of the model, ‘db4’ was the base mother wavelet that yielded the minimum MSE of 0.0043 for the Chittagong station. Thus, this sets the point of reference that is used to compare increases or drops caused by other choices. Table 4 shows that replacing it with ‘db’ resulted in an MSE of 0.0069, which was roughly 60.47% higher than ‘db4’ and indicated that the model’s accuracy was slightly lower. Moreover, with ‘db2’, MSE further increased to 0.0109, 153.49% compared with ‘db4’, which just proves that the performance level significantly decreased, and this would be a bit unsatisfactory for the model performance. Comparatively, ‘db3’ yielded an MSE of 0.0052, corresponding to an increase of 20.93% from ‘db4’. Hence, ‘db3’ is closest to ‘db4’ in terms of accuracy but with a slight sacrifice in performance. The analysis clearly shows that the most optimal wavelet for the Chittagong station is ‘db4’, the second best is ‘db3’, and both ‘db1’ and ‘db2’ give much larger errors. The conclusions drawn from these findings make it abundantly evident that accurate wavelet selection is critical; even little changes in wavelet type can result in a significant difference in forecast accuracy and reliability.

**Table 4:** Impact of different mother wavelet selections on the DWT process.

Station	Mother wavelet	MSE	Influence (%)
Chittagong	db1	0.0069	60.47%
	db2	0.0109	153.49%
	db3	0.0052	20.93%
	db4	**0.0043**	0% (Base)
Dhaka	db1	0.0121	98.36%
	db2	0.0067	9.84%
	db3	0.0069	13.11%
	db4	**0.0061**	**0% (Base)**

Significance value bold.

#### Dhaka station

In the Dhaka station, ‘db4’ gave a minimum MSE of 0.0061, making it the base from which other performances will be compared. In this respect, ‘db4’ gave a minimum MSE of 0.0061, making it the base from which other performances will be compared. For the case of switching to ‘db1’, the MSE rose to 0.0121, representing a rise of about 98.36% about ‘db4’, thus leading to a substantial negative impact on accuracy. With ‘db2’, the MSE was 0.0067, increasing from ‘db4’ by 9.84%, indicating a slight degradation in accuracy. On the other hand, ‘db3’ yielded an MSE of 0.0069, which was higher than `db4` by 13.11%; hence, this wavelet is quite close to `db4` but at a slightly lower precision. This analysis supports that ‘db4’ is the best wavelet for the Dhaka station model as it gives the maximum prediction accuracy among all; in summary, in both stations, ‘db4’ is always the optimum one, which means this one is the optimal choice to maximize accuracy in the models. ‘db3’ is a good alternative, showing just small decreases in accuracy, while ‘db1’ and ‘db2’ involve notably higher error rates, especially over the Chittagong station. These results put into evidence the sensitivity of the model concerning wavelet selection and that even slightly changing the wavelet type may produce effects of great relevance.

The comparison performed in this section gives a deeper insight, confirming that ‘db4’ is still the most effective wavelet in providing good predictions for both stations. Further work can be related to the investigation of larger Daubechies mother wavelets than db4 to establish whether they yield further improvements in predictive accuracy. Extra higher-order wavelets should be embedded in the proposed method of solar radiation prediction, as they may capture finer details, which may perhaps improve the model performance.

### Practical implementations, limitations and future needs

The application of the proposed HP-Filter-DWT-SVM model can be extended toward several practical situations that involve optimization in solar energy systems and climate change mitigation strategies. Due to better accuracy, solar radiation prediction improves the efficiency of the production and distribution systems in solar energy system optimization, thus aligning it with the actual solar availability. Importantly, the model may also contribute significantly to climate change control by making correct forecasts of solar radiation. The accurate solar radiation data from this model is very useful for the improvement of climate models; it helps in the development of renewable systems of energy and promotes adaptation to the climate. Other uses include agricultural planning, whereby the model may provide solar radiation data to help farmers make timely decisions on crop selection and irrigation to enhance productivity and reduce waste of resources. The exact information on solar radiation could also be valuable for urban planning by helping in the design of energy-efficient buildings and urban layouts that maximize natural lighting and, therefore, reduce the reliance on artificial sources of energy. These applications highlight the model’s potential in advancing sustainable energy initiatives, optimizing resource management, and contributing to climate-related research, making it a valuable tool for fostering environmental resilience and supporting long-term sustainability goals.

The analysis was limited to solar radiation data with the DWT of db4 as the mother wavelet, and a sensitivity test for db1-db3 was executed. The research incorporated four detail coefficients and one approximation; hence, it needs improvements to utilize more detail coefficients while capturing a better resolution in the model. Other future works may consider increasing the number of climate variables to be considered for analysis, such as rainfall, temperature, and humidity. Also, other mother wavelets than db4 may be considered in further tuning of the model. It is also recommended that more than four detail coefficients be used to increase the model’s accuracy and resolution. Deep learning techniques could also be applied to increase predictive accuracy. It can also be suggested that advanced techniques like the Multiple Empirical Kernel Learning Machine (MEKLM) technique^64^, the Joint Self-Adaptive Sime Mould Algorithm (JASMA)-based SVM (JASMA-SVM)^65^ , and the Online Sequential Learning Machine (OSLM) be applied for real-time prediction^66^. Development and incorporation of new, more advanced metaheuristic optimization algorithms, such as LCA, PO, HHO, and RIME optimization algorithms, may provide better model parameter tuning and considerably improve overall model performance.

## Conclusion

This research presented a hybrid HP-Filter-DWT-SVM model for the prediction of monthly global solar radiation and outperformed the traditional SVM and hybrid DWT-SVM models by a large margin in terms of prediction accuracy. The HP filter effectively removed low-frequency noise, further enhancing the capability of the DWT to capture the main features of the signal. The research’s key findings, limitations and recommendations are

### Key findings and significance

- The proposed HP-Filter-DWT-SVM model has reduced MSE by 99.76% for Dhaka and 99.77% for Chittagong compared to the traditional SVM model and by 39% and 57%, respectively, compared to hybrid DWT-SVM.
- The model improved 49% in Dhaka and 54% in Chittagong for R^2^ concerning SVM, while 4.40% and 3.16% over hybrid DWT-SVM.
- Such findings are useful for energy production optimization, agricultural planning, and furtherance of climate studies in general, particularly concerning renewable energy generation, efficient land use, and mitigation of climate change. The model enhances the accuracy of predicting solar radiation, which further offers better and more informed decisions about energy grid management, forecasting crop yields, and performing climate modeling. It would not only contribute to sustainability but also place the model as a transformative tool for which wide-ranging implications are considered in policy-making, resource management, and environmental research.

### Limitations

- The investigation was restricted to solar radiation data.
- The DWT considered db4 only as the mother wavelet, where sensitivity analysis has been performed for db1 to db3.
- The study used four detail coefficients and one approximation; it has to be enhanced with more detail coefficients for better resolution of the model.

### Recommendations for future studies

- More climate variables such as rainfall, temperature, and humidity could be integrated.
- Also, test other mother wavelets other than db4 to study further improvements.
- Include more detailed coefficients than four to improve the accuracy and resolution of the model further.
- The model can further leverage deep learning techniques for improved predictive accuracy and a better capability to capture complex patterns in solar radiation data.
- Future studies should proceed by incorporating advanced metaheuristic optimization algorithms, such as LCA, PO, HHO, and the RIME Optimization Algorithm, in carrying out model parameter tuning in an efficient way to further improve the optimization results yielded by the solar radiation prediction models.

In this respect, the study represents an important step ahead in the direction of prediction of solar radiation through helpful advanced techniques of signal processing. By incorporating more variables and refining the model, it holds great promise for climate-related forecasts and supports practical applications in energy and agriculture.

## Data Availability

The data that support the findings of this study are available at Bangladesh Meteorological Department (BMD).
